# Forest ecosystem service functions and their associations with landscape patterns in Renqiu City

**DOI:** 10.1371/journal.pone.0265015

**Published:** 2022-04-06

**Authors:** Yunlu Zhang, Tingting Su, Yue Ma, Yanyinuo Wang, Weiqi Wang, Niyi Zha, Ming Shao

**Affiliations:** School of Landscape Architecture, Beijing Forestry University, Beijing, China; Jinan University, CHINA

## Abstract

Forest ecosystems are crucial to the survival and development of human societies. Urbanization is expected to impact forest landscape patterns and consequently the supply of forest ecosystem services. However, the specific ways by which such impacts manifest are unclear. Therefore, to discuss the relationship between them is of great significance for realizing regional sustainable development. Here, we quantitatively assess the intensity of forest ecosystem service functions and forest landscape patterns in Renqiu City of China’s Hebei Province in 2019 using ArcGIS and FRAGSTATS. We characterize the relationships between forest ecosystem service capacity and landscape patterns, and identify strategies for the spatial optimization of forests. We find that the ecosystem service intensity of forests are significantly correlated with their spatial distribution, forest area ratio, and landscape patterns. Specifically, the percentage of landscape (PLAND) index, landscape shape index (LSI), and contagion (CONTAG) index indices display second-order polynomial relationships with various forest ecosystem service functions, with critical values of 80, 5, and 70, respectively. We propose that forest ecosystem functions can be optimized by optimizing forest landscape patterns. Specifically, to maximize the function of forest ecosystem services, managers should consider the integrity of forest ecosystems, optimize their ability to self-succession, repair service functions of key nodes within forests, enhance forests’ structural stability, optimize forest quality and community structure, and strengthen the efficiency of functional transformation per unit area. Finally, we propose a strategy for the spatial optimization of forests in Renqiu to optimize their associated ecosystem services. This involves protecting important areas for forest ecosystems, rationally organizing different ecological patches such as forests and water bodies to maximize their functions, strengthening the connectivity of scattered forests, and supplementing woodland areas.

## 1 Introduction

Ecosystem services are the benefits that humans derive from ecosystems. In 1997, Costanza and colleagues presented the first quantification of the functional value of the world’s ecosystems [1]. In 2005, the United Nations released the Millennium Ecosystem Assessment System (MESA) [2]. Based on their specific benefits to human society and the natural environment, ecosystem services can be classified under four categories: provisioning services, regulating services, supporting services, and cultural services [3]. Increasingly, researchers in urban planning are using the concept of ecosystem services to understand the impacts of urban spaces on natural systems [4–6].

Forest ecosystems represent important material reserves for the survival and development of human societies [7]. Previous studies have shown that forest ecosystems do not only provide human societies with a variety of ecosystem services (such as wood production and supply, carbon sequestration and oxygen storage, water and soil conservation, and wind and sand control), but also play important roles in supporting ecological communities and maintaining the overall stability of the biosphere.

Human activities and urban expansion increasingly impact forest ecosystems, and consequently undermine the quality of associated ecosystem services [8]. Forested land is often converted for urban use. For instance, 34.05%, 22.58% and 19.65% of the land for new developments in Changsha-Zhuzhou-Xiangtan, the Pearl River Delta and Chengdu-Chongqing, respectively—three major urban areas in China—were converted from forests [9]. In addition to the reduction of forested areas, urban expansion also leads to the degradation of forest ecosystem services [10]. For example, Victoria et al. [11] showed that the destruction of forest ecosystems across cities worldwide threatened only about 4% of the global top 17% priority areas for endangered species. Diaz and Cabido [12] found that a loss of plant diversity, especially of key functional species, affected plant production and decomposition [13], and impaired biogeochemical cycles. Such changes would affect the value of supply services from forest ecosystems. Bala et al. [14] found that a reduction in forested area led to higher surface temperatures and reduced regional precipitation, which in turn reduced evapotranspiration and heat fluxes, affecting energy exchange [15] and water cycling [16]. Similarly, Foley et al. [17] found that a reduction in forested area led to increased soil erosion [18] and river sediment load. Richey [19] found that carbon originally stored in forested land was transported downstream and eventually released as carbon dioxide. In fact, the net carbon emissions from tropical deforestation and degradation in the 21st century almost equal the total carbon emissions from global land use change [20]. Wright and Dobson [21] found that a disruption of forest integrity could trigger changes in the trophic structure of ecological communities, seriously jeopardizing ecosystem stability.

Studies have shown that forests are a major contributor to the value of ecosystem services [22], and that changes in woodlands directly affect the stability and health of both urban and natural systems [23]. The spatial structures of forest ecosystems are impacted by urbanization most directly via changing landscape patterns [24–26]. Urban developments often alter forest landscape patterns by introducing a degree of fragmentation, and changing the sizes of forest patches [27]. For instance, Yuan Wang [28] found that increased urban development led to changes in the spatial patterns of urban forests, an overall decrease in the aggregation of urban forest types, and increases in the number of patches, forest fragmentation, the irregular expansion of boundaries, and changes in forest shape. Marina et al. [29] found that an increase in the area of new urban forest complemented by the addition of more forest patches and further increasing fragmentation. This strengthened the connectivity of existing forests and led to the construction of new types of forest habitat corridors, thereby contributing to the enhancement of existing ecological networks. The specific landscape patterns of urban forests are closely related to urban spatial developments. That is, strong disturbances from human activities can affect urban forest landscape patterns. Furthermore, changes in landscape patterns are likely to affect the integrated functions of forest ecosystems, which in turn affect urban development and ecological stability [30].

An understanding of how changing landscape patterns of forest ecosystems are associated with changing ecosystem service functions can provide for more effective decisions for urban planning. Recent studies have investigated the correlations between the functions of different types of ecosystem services and their landscape patterns. Using multi-criteria evaluation (MCE) and participatory techniques, Rav et al. [31] evaluated the application of different forest cover types in watersheds to test their adaptation to different landscape patterns, and to prioritize areas for forest restoration to improve water-related ecosystem services. Analyzing the effect of wildfire in the Big Sur Forest Ecoregion in California, He et al. [32] showed that wildfires reduced the area, edge density, and isolation of healthy tree patches, and that these did not return to pre-fire levels eight years following the recovery of vegetation. Studying land-cover components and landscape indices, Mladenoff et al. [33] showed that finer-grained data are needed to map within-state ecoregions and discriminate important landscape characteristics. LUDA data, or similar coarse resolution data sources, should be used with caution and the biases fully understood before being applied in regional landscape management.

Most studies investigating changes in forest ecosystem services have focused on changes that have been driven by changes in forest area, as well as on the effects of changes in ecosystem types on ecosystem service functions. In comparison, there has been a lack of in-depth research on the relationship between landscape patterns and changes in ecosystem service functions at the spatial scale. Strategies for enhancing forest ecosystem services through optimizing landscape patterns are also less explored; there are very few theoretical and empirical studies in these areas [34, 35]. Few studies have also addressed the impacts of changing landscape patterns on ecosystem services, which are particularly complex because they tend to vary spatially and geographically [36–41]. To help planners better understand the mechanisms underlying forest ecosystem services, there is a need for research that (i) integrates the natural environmental characteristics and socio-economic features of different study areas, (ii) reveals the responses of forest ecosystem services to changes in landscape pattern indices, and (iii) elucidates the relationships between landscape pattern indices different forest ecosystem service functions. Such work will help to optimize the allocation of forest resources, and to improve efforts for the sustainable development of urban and forest ecosystems [42].

To cope with the impacts of rapid urbanization on ecological habitats, In 2004, China introduced the construction of “national forest cities” under the “National Important Ecological Civilization Construction Strategy” [43]. This study is an exploration of the construction of forest cities, and Renqiu City in Hebei Province is chosen as the object of the study. As one of the first national forest cities in China, it is located in the core of the Beijing-Tianjin-Hebei region and presents significant and clear natural, economic and human characteristics within the study area, which are typical. This study has a clear focus and a better theoretical framework, which can make up for the deficiencies in the current theoretical and methodological research on urban forest planning in China, has important theoretical guidance, and provides practical application value for the construction of forest cities in other regions. In addition, in the past 50 years, countries have carried out theoretical research on urban forest cultivation and management as well as urban forest construction practices with their own characteristics [44–49], such as the Japanese government’s proposal to build "forest cities" [50], the U.S. Forest Service’s urban forestry research program, and some countries and regions in Asia and Africa have also begun to pay attention to urban forestry and carried out different These policies have played a very important role in urban construction, and the development of urban forestry has been recognized worldwide [51]. This study can also provide some theoretical guidance and optimization measures for the same type of cities to promote the sustainable and healthy development of urban forest ecology.

Using a quantitative analysis of spatial patterns, the present study analyzes the impacts of forest landscape patterns on ecosystem services in Renqiu city under different spatial layouts, so as to identify specific layouts and landscape pattern indices that affect the delivery of urban forest ecosystem services. The regular features of forest ecosystem services functions are then summarized to derive a strategy for optimizing forest ecosystem services. Finally, the theoretical basis and technical foundations for the planning, construction, management and sustainable use of urban forests are provided to guide the sustainable development of urban areas.

## 2 Study area

**Fig 1:**
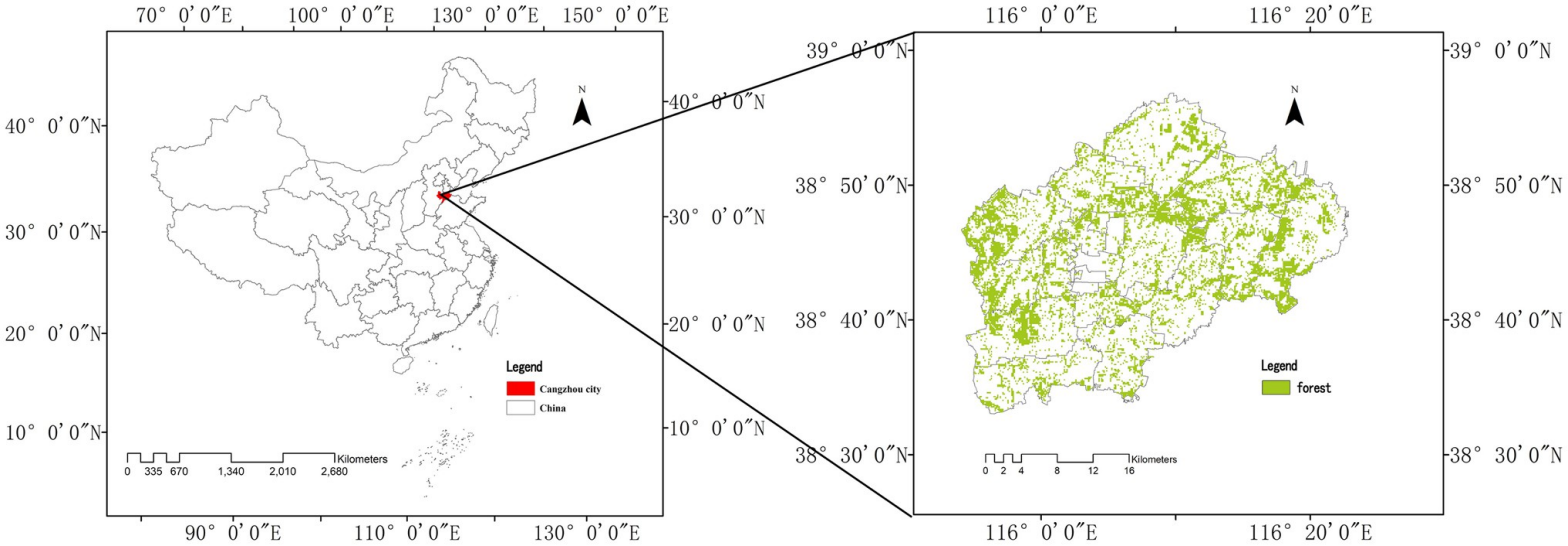
Location map of Renqiu City.

Renqiu City is located in central Hebei Province, on the eastern bank of Lake Baiyangdian (Fig 1). It is adjacent to Wenan County and Dacheng County in the east, Hefei City in the south, the junction of Anxin County and Gaoyang County in the west, and Xiongan New Area in the north. Renqiu City is located in the core area of Beijing-Tianjin-Hebei urban agglomeration, which is the most rapidly developing urban area in China, and belongs to the “economic open belt” around Beijing-Tianjin and the Bohai Sea, while the rapid urbanization development has also caused certain impacts on the urban ecological habitat. As an important pilot city of national forest city construction, Renqiu city has a typical theoretical background and global significance. Renqiu City has a warm temperate semi-humid climate, with four distinct seasons. It is situated within a typical low plain landscape, with depressions scattered throughout the city, making water resources relatively abundant. The wetland area of the city is 0.77 hm^2^, and the existing rivers are the Xiaobai River in the west, the Guyang River in the east, and the Renwen Dry Canal in the middle. The forested area of the city has a coverage of 21.10% and reaches 16466.67 hm^2^, and is located at the forefront of plain counties and cities in Hebei Province.

We delineate “forested land” (Fig 1), as per the definitions of the Chinese Forestry Law and other relevant publications [52, 53], as the category of forest for our study.

## 3 Materials and methods

**Fig 2:**
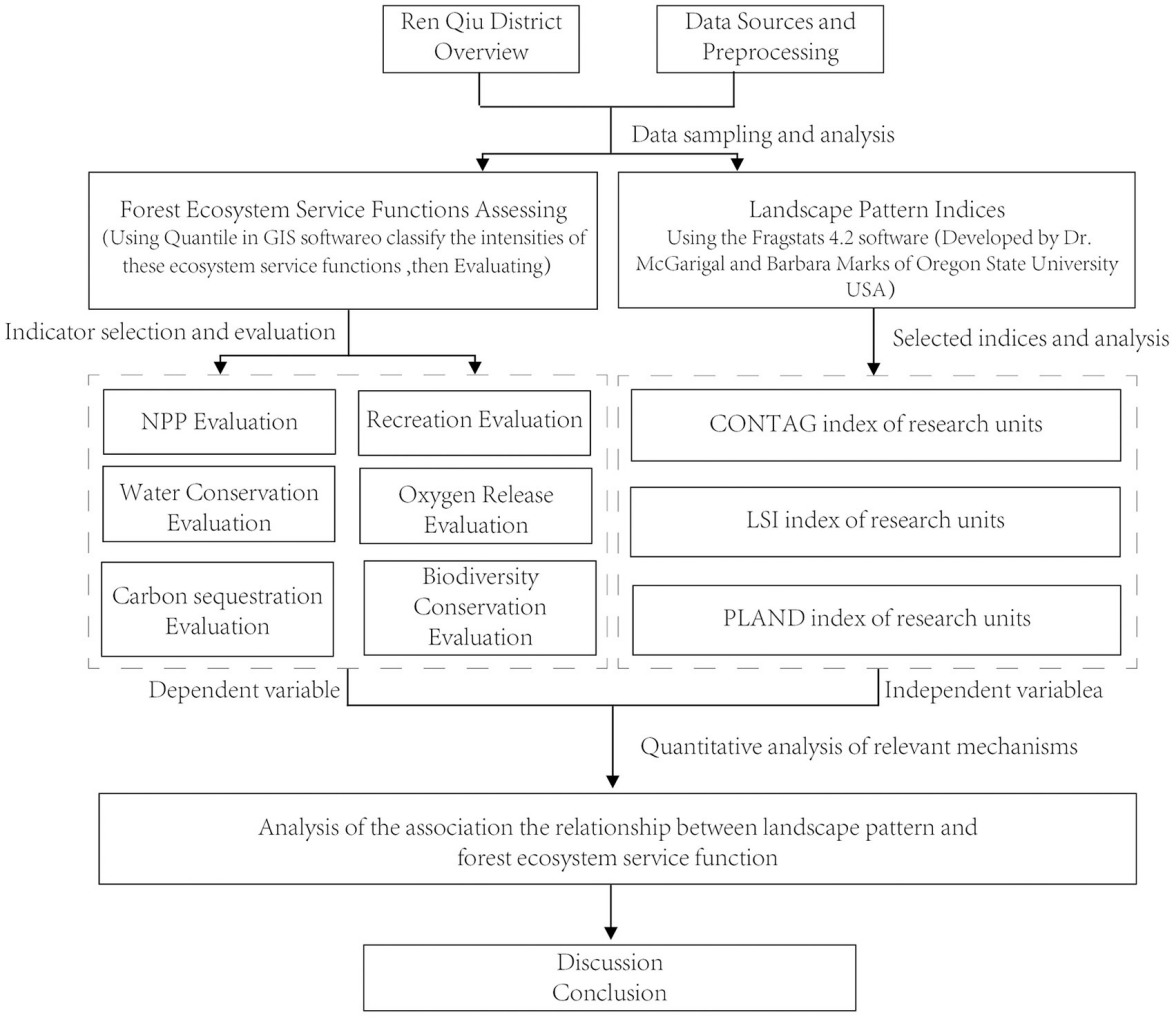
The technical route of the study.

We used ArcGIS 10.2 (ESRI, Redlands, CA, USA) to collect DEM (Data Energy Modernization) data, meteorological data, soil type data, NDVI (normalized vegetation index) data, NPP (net primary productivity of ecosystem) data, land use type data, road and water system data, and related planning data. We then analyzed the data as follows and draw the technical roadmap (Fig 2).

First, we combined the data with the environmental characteristics of Renqiu City, and selected the six indicators of NPP, carbon sequestration, oxygen release, hydrological treatment, biodiversity maintenance, and forest recreation to establish the forest ecosystem service function assessment index system for Renqiu City. Second, we characterized the spatial distribution and composition of forest ecosystem services in Renqiu City using three landscape pattern indices: the percentage of landscape area occupied by patches (PLAND), the landscape shape index (LSI) and the forest sprawl index (CONTAG). Third, we investigated the relationships between landscape patterns and forest ecosystem services using regression analyses, with the landscape pattern indices PLAND, LSI and CONTAG as independent variables and the six indicators of four major forest ecosystem service functions as dependent variables. We used the SPSS software (version 11.01; SPSS Inc., Chicago, IL, USA for statistical analyses.

### 3.1 Data sources

The data used in the study mainly include DEM (Data Energy Modernization) data, meteorological data, soil type data, NDVI (normalized vegetation index) data, NPP (net primary productivity of ecosystem) data, land use type data, road and water system data, and related planning data. The elevation and remotely-sensed data (NPP, NDVI) were sourced from the National Geographic Information Spatial Data Cloud Platform (http://www.gscloud.cn/); the meteorological data (i.e., temperature, rainfall, soil, and land use) were sourced from the Resource and Environment Science Data Center of the Chinese Academy of Sciences (www.resdc.cn); while the POI (Point of Interest) vector data of basic transportation facilities were sourced from the World Data Map (www.openstreetmap.org) (Table 1). All spatial data were resampled to a 30×30 m raster in ArcGIS 10.2 (ESRI, Redlands, CA, USA), and the coordinate system was standardized.

**Table 1:** Data types and sources.

Serial number	Data Type	Data source	Data year
**1**	NPP	National Geographic Information Spatial Data Cloud Platform	2018
**2**	Land Use Type	Resource and Environment Science and Data Center of Chinese Academy of Sciences	2018
**3**	Elevation/Slope	National Geographic Information Spatial Data Cloud Platform	——
**4**	Road/Water System	World Data Map	2018
**5**	Soil Infiltration Factor	Resource and Environment Science and Data Center, Chinese Academy of Sciences	2009
**6**	Average Rainfall	Resource and Environment Science and Data Center, Chinese Academy of Sciences	2018
**7**	Average Temperature	Resource and Environment Science and Data Center, Chinese Academy of Sciences	2018
**8**	NDVI	National Geographic Information Spatial Data Cloud Platform	2018

### 3.2 Research methodology

#### 3.2.1 Assessing forest ecosystem service functions

Forest ecosystem service functions refer to natural environmental conditions and ecological processes that form and maintain the utility of forest ecosystems on which human beings depend [54, 55]. According to China’s national Forest Ecosystem Service Function Assessment Specification (GB/T 38582–2020) standard, forest ecosystem service functions include supply services, regulating services, supporting services, and cultural services [56–60]. The present study on forest resources of Renqiu City applies this classification framework of ecosystem services, reliable assessment indicators, availabile data and relevant research. In addition to the environmental characteristics of Renqiu City, we selected six indicators to assess the city’s forest ecosystem service functions; these include net primary productivity (NPP), carbon sequestration, oxygen release, hydrological treatment, biodiversity maintenance, and forest recreation (Table 2).

**Table 2:** Forest ecosystem service function indicators.

Service Type	Functional indicators
Supply Services	NPP (net primary productivity)
Regulating Services	Carbon sequestration, oxygen release, water retention
Supporting Services	Biodiversity conservation
Cultural Services	Forest recreation

① Net primary productivity (NPP)
  NPP is the amount of primary production (i.e., the amount of energy fixed or organic matter produced per unit area per unit time) that is left after subtracting for the energy or organic matter used in plant respiration. NPP is usually estimated by field measurements and model calculations that have shown to be reliable [61–64]. We used data on NPP (measured in g/m^2^) based on the light energy utilization model, GLO_PEM [65].
$$NPP=PAR\times FPAR\times \varepsilon \left(x,t\right)-R_{a}$$ (1)
  Where *PAR* is photosynthetically active radiation, *FPAR* is the ratio of photosynthetically active radiation absorbed by vegetation, *ε* is based on the GPP (Gross Primary Productivity) concept of actual light energy utilization, and *R*_*a*_ is vegetative autotrophic respiration (which includes maintenance respiration, *R*_*m*_, and growth respiration, *R*_*g*_).
② Carbon sequestration and oxygen release
  In forest ecosystems, carbon sequestration and oxygen release refer to the exchange of oxygen and carbon dioxide that occur via the photosynthesis of green plants, which fix and reduce carbon dioxide in the atmosphere while releasing oxygen [66].
$$G_{v}=1.63R_{c}*A*NPP;G_{0}=1.19A*NPP$$ (2)
  Where *G*_*v*_ is the annual carbon sequestration by vegetation (in g/a); *G*_0_ is the annual oxygen release from the ecological space (in g/a); 1.63 and 1.19 are calculated coefficients, respectively, from the fact that plants can fix 1.63 g of carbon dioxide and release 1.19 g of oxygen for every 1 g of dry matter produced; *R*_*c*_ is the content of carbon in CO_2_, which takes the value of 27.27%; *A* is the area of ecological space (in hm^2^); and NPP is the mean net primary productivity of vegetation (in g/m^2^).
③ Water conservation
  Water harvesting refers to the interaction between forest ecosystems and water, which intercepts, infiltrates, and accumulates precipitation, and regulates water flow and water cycling through evaporation. Key areas that can assume the function of water conservation in the present and future are identified based on the index [67]:
$$WR={NPP}_{mean}*F_{sic}*F_{pre}*(1-F_{slo})$$ (3)
  Where *WR* is the index summarizing the capacity of the ecological space to support water, *NPP*_*mean*_ is the mean value of net primary productivity, *F*_*sic*_ is the soil infiltration factor, *F*_*pre*_ is the average precipitation factor, and *F*_*slo*_ is the slope factor. The values of *F*_*sic*_, *F*_*pre*_, and *F*_*sio*_ are normalized between 0 and 1 according to the maximum-minimum method.
④ Biodiversity conservation
  Biodiversity conservation is one of the most important supporting ecosystem services in forests. The current and future capacity of different regions to provide biodiversity conservation can be identified by the indicator [68]:
$$Sblo={NPP}_{mean}*F_{pre}*F_{tem}*(1-F_{alt})$$ (4)
  Where *Sblo* is the Biodiversity Conservation Service Capacity Index, *NPP*_*mean*_ is the mean value of net primary productivity, *F*_*pre*_ is the average rainfall, *F*_*tem*_ is the average temperature, and *F*_*alt*_ is the elevation factor. The values of *F*_*pre*_, *F*_*tem*_, and *F*_*alt*_ are normalized between 0 and 1 according to the maximum-minimum method.
⑤ Recreation
  Recreation is an important aspect of forest ecosystem services. Forests provide leisure, entertainment, and aesthetic enjoyment for people. The capacity of forest ecosystems to provide cultural services is determined by evaluating multiple indicators that correspond to three aspects of recreation in forests: recreational resources, recreational facilities, and recreational locations [69].
$$S=\sum_{i}^{n}WiXi$$ (5)
  where *S* is the comprehensive index of recreation use, W_i_ is the recreation factor rank value, X_i_ is the weight of different recreation factors, and n is the number of evaluation factors.

#### 3.2.2 Landscape pattern indices

Landscape pattern indices greatly condense information about the spatial structures of ecosystems. Here, we use landscape pattern indices to establish quantitative indicators of the association between ecosystem services functions and forest spatial structure. There are approximately 200 different landscape pattern indices, however, some of them are highly correlated with one another and therefore have problems with redundancies. Findings from previous studies [70–73] suggest that the spatial inventory and spatial shape of an ecosystem are the core indicators for characterizing its spatial characteristics. Based on the landscape characteristics of Renqiu City, we calculated the percentage of landscape area occupied by patches (PLAND) and derived the landscape shape index (LSI).

① PLAND corresponds to the percentage of patches in the landscape, and thus is one of the bases for determining the matrix or dominant element in the landscape. This makes it an important factor for determining ecosystem indicators such as biodiversity and the number of dominant species in the landscape. PLAND is calculated according to
$$P_{i}=\sum_{j=1}^{n}a_{ij}/A\left(100\right)$$ (6)
  where *P*_*i*_ is the proportion of the landscape occupied by the patch type, *a*_*ij*_ is the plot area (m^2^), and *A* is the total landscape area [74].
② LSI, the shape index of patches in the landscape, is measured by the deviation in the shape of a patch from a circle or a square of the same area, so as to show the complexity of the shape. LSI is calculated according to
$$LSI=\frac{0.25TE}{\sqrt{TA}},$$ (7)
  where TE is the total length of all patch boundaries in the landscape, and TA is the total area of the landscape [75].
③ The forest sprawl index (CONTAG) is an information-theoretic-based index that measures the degree of spatial aggregation, or the tendency for different patch types in the landscape to expand. Since this index contains spatial information, it is one of the most important indices for describing landscape patterns. CONTAG is calculated according to
$$CONTAG=\left[1+\sum_{i=1}^{m}\sum_{j=1}^{m}p_{ij}\;\text{ln}\left(p_{ij}\right)/2\;\text{ln}\left(m\right)\right]\left(100\right)$$ (8)
  where *p*_*ij*_ is the probability that two randomly selected adjacent pixels belong to patch types *i* and *j*, and *m* is the total number of patch types in the landscape [76].

After selecting the landscape pattern indices, we analyzed the landscape patterns using the FRAGSTATS 4.2 (Oregon State University, Corvallis, OR, USA) software to characterize the landscape features of forest ecosystem services in Renqiu City in terms of their spatial distribution and compositional configuration.

#### 3.2.3 Investigating the relationship between landscape pattern and forest ecosystem service function

**Fig 3:**
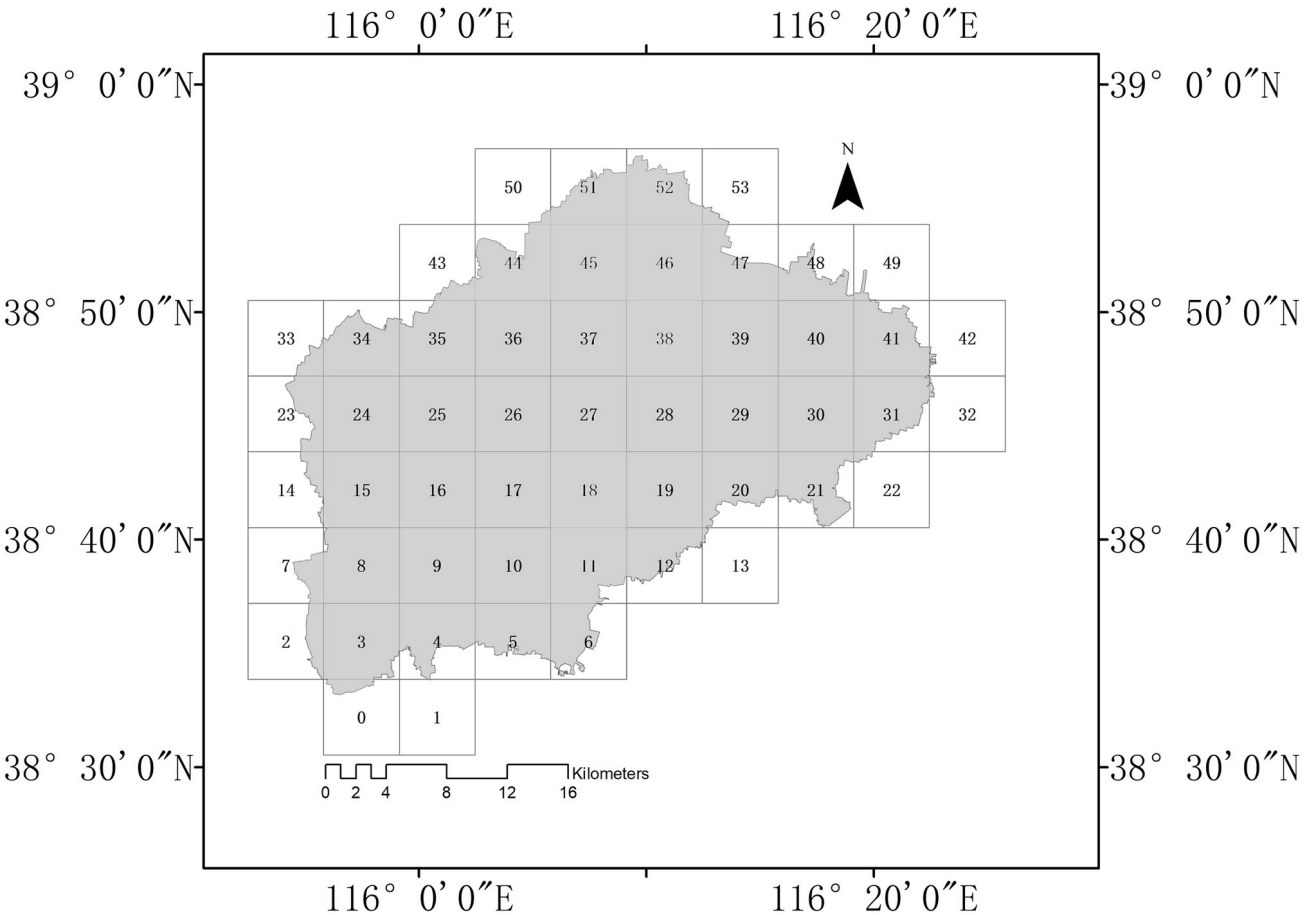
Grid-based division of Renqiu City into 53 individual cells.

To investigate the relationship between landscape patterns and forest ecosystem service functions in Renqiu City, we divided the city uniformly using a 5000×5000 m grid in ArcGIS 10.2 software to yield a total of 53 cells (Fig 3). This grid-based division can eliminate the influence of human management and natural features, thus unifying the overall area, pattern, and characteristics of the study cells, and avoiding biases in the results.

We conducted a regression analysis in the statistical package SPSS software (version 11.01; SPSS Inc., Chicago, IL, USA), using the landscape pattern indices PLAND, LSI, and CONTAG as independent variables, and the six indicators of four major ecosystem service functions as dependent variables. The curve fitting models used in the regression analysis mainly included the fitted linear equation, the fitted quadratic equation, the fitted composite curve model, the fitted logarithmic equation, the fitted exponential equation, the fitted multiplied power curve model, and the fitted logistic curve model. The best fitted equation was selected based on the magnitude of the coefficient of determination (R^2^) as well as a significance test.

## 4 Results

### 4.1 The varying intensity of forest ecosystem service functions across space

**Fig 4:**
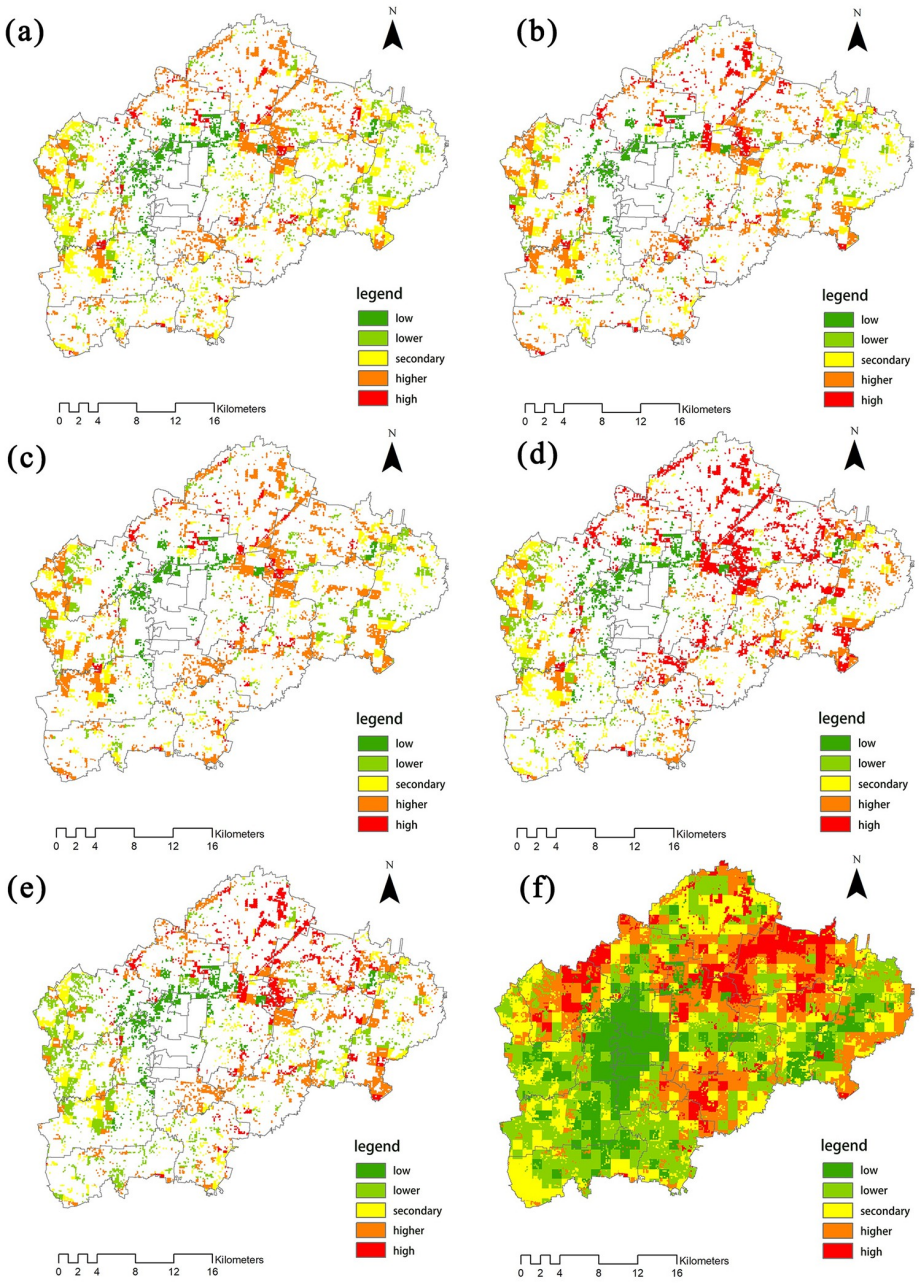
Spatial distribution of forest ecosystem service function intensity. Maps show the distributions of (a) the intensity of NPP, (b) the intensity of carbon sequestration, (c) the intensity of oxygen release, (d) the intensity of water conservation, (e) the intensity of biodiversity conservation, and (f) the intensity of recreation.

Using the methods for evaluating ecosystem service functions mentioned above, we conducted separate single-factor evaluations for the intensity of NPP, carbon sequestration, oxygen release, water conservation, biodiversity conservation, and recreation in the study area. The results of the evaluations are shown in Fig 4. We used six single-factor classification methods to classify the intensities of these ecosystem service functions in GIS software using Quantile (quantile). We then classified the normalized values of functional intensity into five levels, ordered from low to high: “lower,” “low,” “medium,” “high,” and “higher.”

#### 4.1.1 NPP

In Renqiu City, areas with high values of NPP service capacity (Fig 4A) are mainly distributed around the rivers (698.39 g/m^2^), in the north of the Beixinzhuang Township (725.64 g/m^2^), the Yucun Township (763.48 g/m^2^), the Liangzhao Township (783.26 g/m^2^), as well as the southern part of the city (809.95 g/m^2^). These areas have dense networks of rivers and canals, large forested areas, and thus relatively high average annual NPP. In contrast, areas with low NPP service capacity are mainly located in the northern (64.72 g/m^2^) and northeastern (139.06 g/m^2^) parts of the city. Differences in NPP are most likely due to differences in plant species composition, respiration rates, nutrients, and water.

#### 4.1.2 Carbon sequestration and oxygen release

The high values of carbon sequestration and oxygen release capacity (Fig 4B and 4C) are directly related to the high values of NPP. Areas with high carbon sequestration and oxygen release capacity are mainly located at the junction of the Renwen Gan Canal and the Guyang River, as well as along the Guyang River and the area bordering Goyang County in the east. Levels of carbon sequestration for these areas range from 155.31 × 10^7^ g to 4.18 × 10^7^ g, while levels of oxygen release range from 1.12 × 10^8^ g to 34 × 10^8^ g. These are also areas with high NPP service capacity.

#### 4.1.3 Water conservation

Areas with the highest values of water conservation capacity (Fig 4D) are distributed widely across the northern part of Renqiu City (6.39 × 10^−2^) as well as the southeastern part of the city (8.32 × 10^−2^). These areas are adjacent to Lake Baiyangdian, and are densely comprised of rivers and canals. High values of water conservation capacity are also scattered across the east and south of Renqiu City; here, the highest value (8.74 32 × 10^−2^) is found at the junction of the Renwen trunk canal and the Guyang River. The high vegetation cover in this region plays an important role in regulating and conserving water resources.

#### 4.1.4 Biodiversity conservation

High values of biodiversity conservation capacity (Fig 4E) are mainly found in the northern (6.58 × 10^−2^), southern (7.73 × 10^−2^), and southeastern (8.38 × 10^−2^) parts of Renqiu City. These patterns reflect the distribution of water conservation capacity throughout the city. This is due to the strong NPP capacity in these areas, and the dense networks of rivers and canals. Furthermore, lakes serve as important sites for supporting plant and animal populations, as well as for the production of biomass and energy; they thus have an irreplaceable role in biodiversity conservation.

#### 4.1.5 Recreation

We evaluated the recreational capacity forests in Renqiu City (Fig 4F) based on factors such as elevation, slope, vegetation cover, land type, roads, and water systems. High values of recreational capacity are concentrated in the Qingta Township (0.725), the area of the Beixinzhuang Township that is adjacent to Lake Baiyangdian (0.793), most areas in Liangzhao Township (0.806), the southern part of Yuxiang Township (0.839), the northern and southern parts of Yulunbu Township (0.846), most areas in Majiawu Township (0.887), and the northwestern parts of Beihan Township (0.901). These areas are all distantly located from the urban center of the city, and generally reflect the distribution of forests in the region. These results indicate that areas in Renqiu City that are proximal to countryside forests and water sources have high recreational capacities, and that the development of projects involving cultural services can be carried out at these areas in the future.

### 4.2 Forest ecosystem service functions within study units

**Fig 5:**
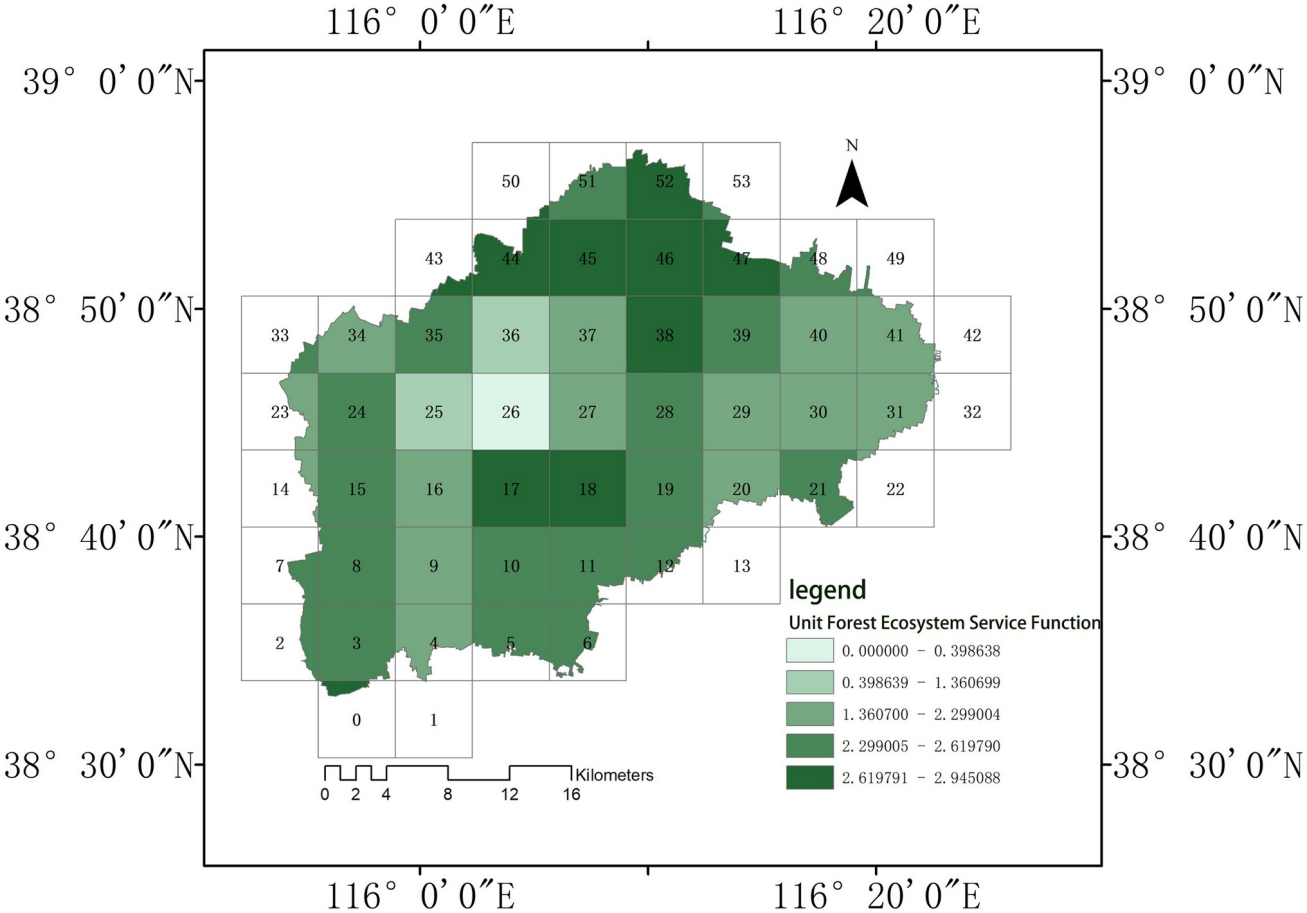
Distribution of forest ecosystem service functions across the study units.

We divided forest ecosystem functions into five levels using the natural breakpoint method [77–79]. Table 3 and Fig 5 show the levels of forest ecosystem service functions within study units in Renqiu City.

**Table 3:** Forest ecosystem service functions of the study units.

Number	Supply Services	Regulating Services			Supporting Services	Cultural Services
	NPP (g/m^2^·a)	Carbon Sequestration (g)	Oxygen Release (g)	Water Conservation	Biodiversity Conservation	Recreation
**0**	605.1749519	25618211428	67864737072	0.05261308	0.05255458	0.53122139
**1**	0	0	0	0	0	0
**2**	547.2499506	17460625224	46272147856	0.04630379	0.04676639	0.53611573
**3**	528.4954485	92653902070	249222072620	0.04581906	0.04621796	0.51373846
**4**	465.0790814	81895608789	218164081676	0.04296537	0.04194242	0.45866731
**5**	530.1084946	60262555402	159782838096	0.05088988	0.04962202	0.49525308
**6**	534.5308209	99580514814	267211375904	0.05295014	0.05152753	0.48521463
**7**	588.0586744	5999704128	16062164904	0.04981168	0.05038504	0.48586448
**8**	534.3904951	264314381250	706244093944	0.04678342	0.04711433	0.49074833
**9**	403.6087862	96843805970	259863013730	0.03666426	0.03638576	0.42512784
**10**	524.9022594	111053028318	297163319464	0.05113119	0.04973745	0.46478112
**11**	560.1260363	118499459424	318134975176	0.0560459	0.05466314	0.60482258
**12**	499.5174283	22764051394	60920381512	0.05178744	0.05024737	0.58500569
**13**	0	0	0	0	0	0.55266974
**14**	507.1252597	58908028666	156040191280	0.04471713	0.04418228	0.47991643
**15**	501.5650235	272682531732	723244907788	0.04418523	0.04438264	0.44087455
**16**	384.2612271	123092591319	328176758792	0.03492095	0.03497385	0.41412485
**17**	551.4125636	60383055470	157170783312	0.05463847	0.05310058	0.43158466
**18**	593.085158	185066037548	496751875756	0.06103107	0.05923384	0.63754434
**19**	531.967067	135978930842	366776057288	0.0566572	0.05487395	0.55659666
**20**	479.7436768	155068096506	416930509440	0.05262346	0.05089379	0.44050025
**21**	537.0980012	243914799182	650998780464	0.06102126	0.05907837	0.51122214
**22**	438.964323	12780666084	34189815800	0.05015857	0.04853767	0.60294096
**23**	483.0828899	129324943176	347230826672	0.04337316	0.04214344	0.51088514
**24**	484.9666884	231272728262	625175428452	0.04442443	0.0431153	0.6185038
**25**	192.1872121	59071569034	157371220602	0.01730973	0.0171602	0.50616393
**26**	31.46640524	533809238	1415402230	0.00214046	0.0020768	0.34944408
**27**	343.2124915	49557229530	129857455892	0.0351335	0.03405999	0.53563133
**28**	526.7931488	208401524694	562555695916	0.0570901	0.05513405	0.50584992
**29**	449.3928555	106349648430	283592914076	0.05006778	0.04832947	0.55341107
**30**	451.2594037	193397785017	513119291464	0.05177895	0.04996763	0.44757745
**31**	425.5461676	40147223226	104166144676	0.04940259	0.04785203	0.55701561
**32**	0	0	0	0	0	0.64581982
**33**	530.4358216	55781785338	149809414648	0.04796373	0.04625576	0.50144988
**34**	472.8760763	183347467980	487977485088	0.04347905	0.04201687	0.64062478
**35**	478.4586391	81469857143	220843227444	0.0467359	0.04503134	0.67947179
**36**	249.0074801	93985904553	253372197676	0.02466554	0.02391308	0.54320541
**37**	313.4492554	173679123426	467748785664	0.03275308	0.03163534	0.67324439
**38**	561.9882438	383377286362	1025192805680	0.06150784	0.05933003	0.69295513
**39**	500.3917844	144410770762	389859894980	0.05673414	0.05462968	0.62586561
**40**	450.3524788	254822306627	684264838180	0.05226617	0.05048293	0.5943212
**41**	432.7035444	133434833936	357268206240	0.05132929	0.04959053	0.49097551
**42**	0	0	0	0	0	0.63873259
**43**	617.3821544	13036066184	37025906256	0.06161651	0.0594357	0.6846151
**44**	585.5225343	131627689898	350853111372	0.06029974	0.05807143	0.5818961
**45**	583.3699164	86703697841	231718804020	0.06219044	0.05989279	0.54905036
**46**	606.2014029	225044853236	601734889384	0.06745874	0.06490449	0.58826609
**47**	578.6979519	103167044030	277514455636	0.06588765	0.06360663	0.71465242
**48**	495.1577488	49288761286	130136474120	0.05826489	0.0560594	0.69694325
**49**	474.4250153	1985045916	5509334200	0.05617894	0.05429585	0.56862748
**50**	599.9567068	16383131848	43753903872	0.06376777	0.06109522	0.56700176
**51**	566.9465927	82468734295	222008968294	0.06123046	0.05891063	0.53517997
**52**	593.7171579	126216975174	339302383780	0.06713701	0.06449155	0.57190677
**53**	509.276825	1718982440	4523339920	0.05781247	0.05579591	0.55011728

The highest levels of forest ecosystem service function in Renqiu City are found in the northern parts of the city (units Z39, Z45, Z46, Z47, and Z52), where their values peak at 2.95; this is followed by the southeast parts of the city (units Z17 and Z18) and areas near the three water systems in Renqiu City (units Z3, Z8, Z12, Z15, Z19, Z24, Z28, Z35, and Z39). The central urban areas (units Z25, Z26, and Z36) have a lower level of forest ecosystem service function, at values of approximately 0.40. Levels of forest ecosystem service function can vary by over 737% across Renqiu City.

**Fig 6:**
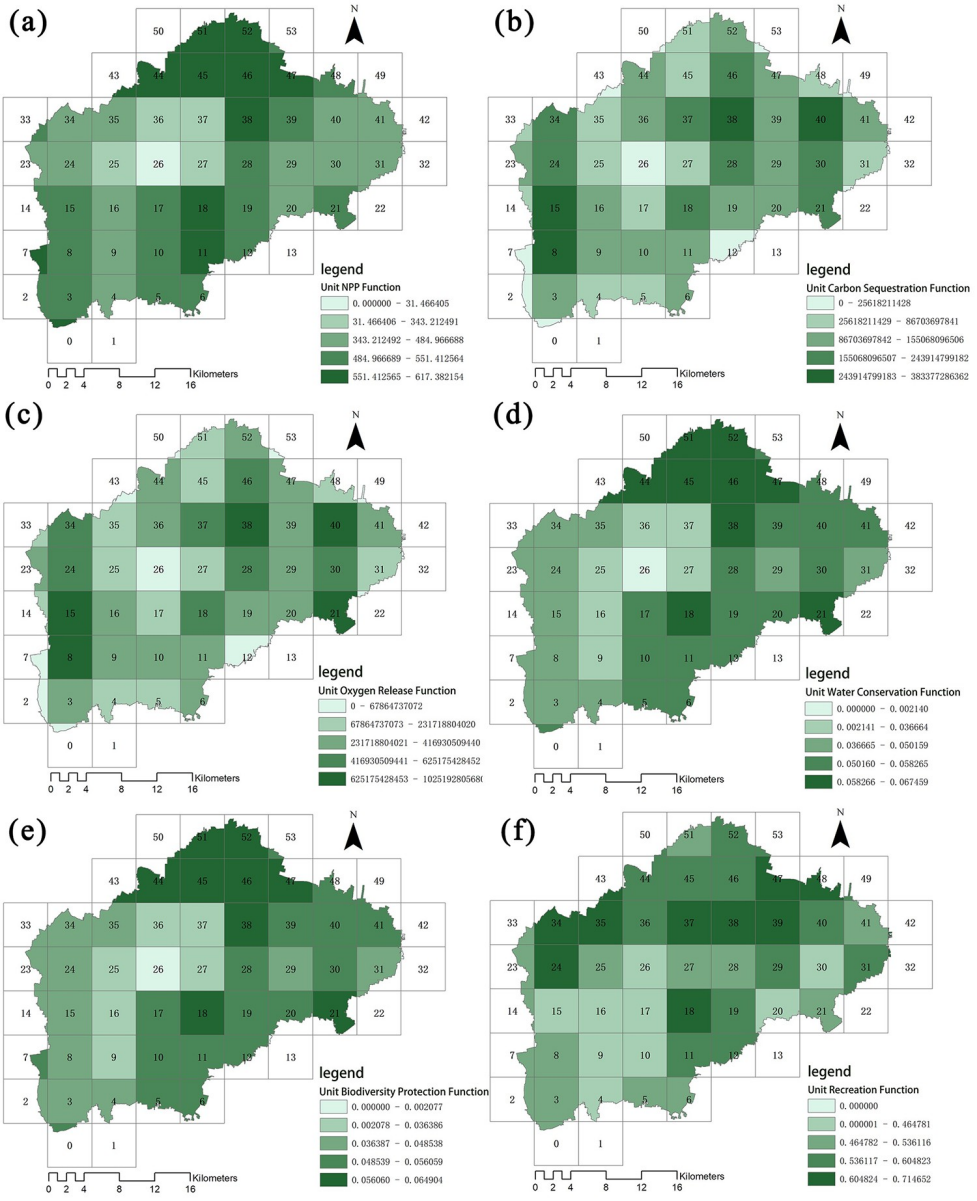
Forest ecosystem service functions across study units. Maps show the distributions of (a) NPP, (b) carbon sequestration, (c) oxygen release, (d) water conservation functions, (e) biodiversity conservation and (f) recreation.

Differences in NPP service capacity can vary by over 1960% in Renqui City (Fig 6A). NPP is at its highest intensity in the north of Renqiu City (units Z38, Z44, Z45, Z46, Z47, and Z52), and peaks at 617.38 g/m^2^·a here. In contrast, the central part of the city (unit Z26) has the weakest relative service capacity (31.47 g/m^2^).

Carbon sequestration services (Fig 6B) and oxygen release services (Fig 6C) are highest at the junction of the Xiaobai River and the Renwen Dry Drainage Basin (units Z8, Z15, Z38, and Z40), where carbon sequestration peaks at 2.44 × 10^11^ g and oxygen release peaks at 3.83 × 10^11^g. In contrast, the central part of the city (unit Z26) has the lowest carbon sequestration (2.56 × 10^10^g) and oxygen release (6.78 × 10^10^g). These differences in levels of carbon sequestration and oxygen release across Renqiu City correspond to differences of over 953% and 565%, respectively.

High value areas for water conservation (Fig 6D) are concentrated in the northern parts of Renqiu City (units Z18, Z20, Z38, Z44, Z45, Z46, Z47, Z51, and Z52) as well as in the northern parts of the Wengan Canal, near the Guyang River; water conservation capacity peaks at 6.75 × 10^−2^ in these locations. In contrast, the central part of Renqiu City has the lowest capacity for water conservation (3.67 × 10^−2^). Water conservation capacity varies by over 183% across Renqiu City.

The distribution of supporting services (Fig 6E) reflects the distribution of forests in Renqiu City. Areas with higher proportions of forest have higher service capacities due to better natural conditions. Values of supporting services peak at 6.49 × 10^−2^ in these areas and are approximately 3247% higher than the lowest values.

The areas with high intensities of cultural services (Fig 6F) are distributed along the Renwen trunk canal and the adjacent Baiyangdian area (unit Z24, Z34, Z35, Z37, Z38, and Z39). Cultural services peak at 0.71 here and are 154% higher than the lowest value in Renqiu City. These areas of high cultural service capacities can significantly contribute to the overall forest ecosystem service capacity of Renqiu City as a whole.

### 4.3 The relationship between landscape pattern and forest ecosystem service function in Renqiu City

We converted the 53 study units into an Arc Grid format, and calculated the values of the PLAND, LSI and CONTAG for forest ecosystems in the 53 study units using the landscape pattern analysis software FRAGSTATS 4.2 (Developed by Dr. McGarigal and Barbara Marks of Oregon State University USA). The results are shown in Tables 4–6. Units Z30, Z33, Z46, and Z6 have the highest PLAND values of 99.5556, 97.4026, 96.8085, and 96.6667, respectively, indicating that these areas have higher proportions of forest ecosystems, and are thus the dominant ecosystems in the region. Units Z29, Z19, Z27, and Z3 have the largest LSI values, with values of 8.8788, 8.8125, 8.5556, and 8.5, respectively, indicating that these areas are rich in shape variation, less subjected to human interference, and more concentrated in distribution. Units Z30, Z46, Z33, and Z20 have the largest CONTAG values with values of 96.4149, 89.2536, 88.8604, and 88.7142, respectively, indicating that these areas support a high degree of aggregation of dominant forest patches, and good connectivity.

**Table 4:** PLAND values of individual study units.

Number	PLAND	Number	PLAND	Number	PLAND	Number	PLAND	Number	PLAND
**0**	87.8788	**11**	83.2487	**22**	100	**33**	97.4026	**44**	77.551
**1**	0	**12**	55	**23**	92.887	**34**	91.018	**45**	62.1849
**2**	45.6522	**13**	0	**24**	89.7119	**35**	76.8116	**46**	96.8085
**3**	87.037	**14**	76.2887	**25**	52.7536	**36**	74.321	**47**	90.1734
**4**	89.1192	**15**	95.4365	**26**	72.8395	**37**	89.8687	**48**	90.7563
**5**	70.4918	**16**	57.4405	**27**	79.2035	**38**	91.6528	**49**	100
**6**	96.6667	**17**	77.7293	**28**	89.9425	**39**	92.1824	**50**	100
**7**	82.1429	**18**	80.6122	**29**	95.3901	**40**	93.4043	**51**	85.4701
**8**	95.8128	**19**	93.609	**30**	99.5556	**41**	87.1901	**52**	89.5028
**9**	72.8448	**20**	96.0452	**31**	57.6271	**42**	0	**53**	100
**10**	75.8242	**21**	94.6512	**32**	0	**43**	100		

**Table 5:** LSI values of individual study units.

Number	LSI	Number	LSI	Number	LSI	Number	LSI	Number	LSI
**0**	2.4545	**11**	7.7692	**22**	1.6	**33**	3.4444	**44**	7.7143
**1**	0	**12**	3.7	**23**	4.3333	**34**	7.7429	**45**	6.2222
**2**	2.4	**13**	0	**24**	6.881	**35**	7.0385	**46**	5.6765
**3**	7.25	**14**	4	**25**	8.2222	**36**	7.8857	**47**	6.56
**4**	7.2222	**15**	7.8864	**26**	5.7727	**37**	7.5	**48**	5.7619
**5**	5.4211	**16**	7.8929	**27**	8.5556	**38**	7.8723	**49**	1.5
**6**	5.36	**17**	8.3333	**28**	6.8889	**39**	8.5	**50**	1.6667
**7**	3.5	**18**	7.871	**29**	8.8788	**40**	7.381	**51**	5.15
**8**	5.725	**19**	8.8125	**30**	6.1163	**41**	6.3	**52**	4.3462
**9**	6.8846	**20**	6.7568	**31**	4.9412	**42**	0	**53**	2
**10**	7.9583	**21**	5.1951	**32**	0	**43**	7.7143		

**Table 6:** CONTAG values of individual study units.

Number	CONTAG	Number	CONTAG	Number	CONTAG	Number	CONTAG	Number	CONTAG
**0**	67.0442	**11**	56.2886	**22**	0	**33**	88.8604	**44**	58.5828
**1**	0	**12**	20.0131	**23**	83.3963	**34**	70.5874	**45**	37.5977
**2**	34.0855	**13**	0	**24**	71.139	**35**	49.0721	**46**	89.2536
**3**	67.5208	**14**	28.0764	**25**	32.3548	**36**	47.8555	**47**	74.0127
**4**	66.4716	**15**	83.2989	**26**	45.4661	**37**	72.062	**48**	69.818
**5**	50.581	**16**	30.5833	**27**	47.6059	**38**	74.6509	**49**	0
**6**	87.9432	**17**	41.5388	**28**	73.2197	**39**	73.9034	**50**	0
**7**	40.2283	**18**	54.4865	**29**	81.1396	**40**	79.9635	**51**	73.3847
**8**	87.2789	**19**	78.8272	**30**	96.4149	**41**	68.8753	**52**	79.8718
**9**	46.293	**20**	88.7142	**31**	30.5659	**42**	0	**53**	0
**10**	51.9064	**21**	83.622	**32**	0	**43**	0		

#### 4.3.1 Relationship between the forest landscape percentage (PLAND) index and forest ecosystem service function

**Fig 7:**
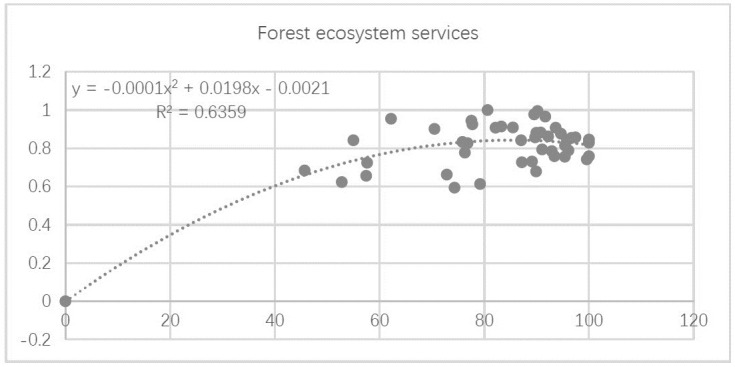
Relationship between forest landscape percentage and forest ecosystem service capacity.

The relationship between the PLAND index and forest ecosystem service function for the 53 study units is shown in Fig 7. Forest ecosystem service function increased polynomially in the second order with increasing values of PLAND. Forest ecosystem service function capacity increased rapidly when PLAND values ranged between 40–60.

**Fig 8:**
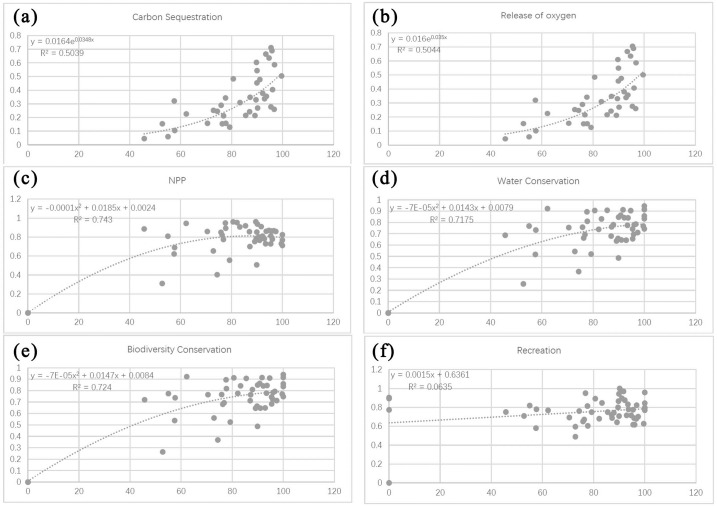
Relationships between the forest landscape percentage index (PLAND) and four ecosystem service functions. Plots show the relationship between PLAND and (a) carbon sequestration capacity, (b) oxygen release capacity, (c) NPP capacity, (d) water conservation capacity, (e) biodiversity conservation capacity, and (f) recreation capacity.

Based on the fitted curve in the regression, the relationship between the indicators of forest ecosystem services and the PLAND index can be classified into three categories: (1) a significant exponential relationship, as shown between PLAND and carbon sequestration capacity and oxygen release capacity (Fig 8A and 8B); (2) a significant second-order polynomial relationship, as shown between PLAND and capacities for NPP, water conservation, and biodiversity conservation (Fig 8C–8E); and (3) no significant association, as shown between PLAND and recreational capacity (Fig 8F).

The first group of results show a clear exponential relationship between carbon sequestration and oxygen release capacity with increasing percentage of forest area. In particular, there is a rapid increase in capacities for these two services at PLAND values approximating 80% (Fig 8A and 8B).

The second group of results show that capacities for NPP, water conservation, and biodiversity conservation increase with increasing PLAND, in a significant second-order polynomial relationship. The capacities of these three functions increase significantly when PLAND values lie between 40% and 60%, and gradually plateau as PLAND increases to 80% (Fig 8C–8E).

The third group of results have poor fit (i.e., R^2^ approximates zero). They indicate that recreational capacity varies irregularly with PLAND, making it difficult to predict its changing behavior using a simple or segmented function (Fig 8F).

In sum, the results show that the forest landscape percentage index (PLAND) has a significant exponential relationship with forest ecosystem service functions, and that ecosystem service capacity increases with increasing percentage of forest area such that when forest percentage approximates the critical value of 80, ecosystem service capacity tends to stabilize owing to the forest ecosystem’s inherent stability and regulation capacity.

#### 4.3.2 Relationship between landscape shape index (LSI) and forest ecosystem service function

**Fig 9:**
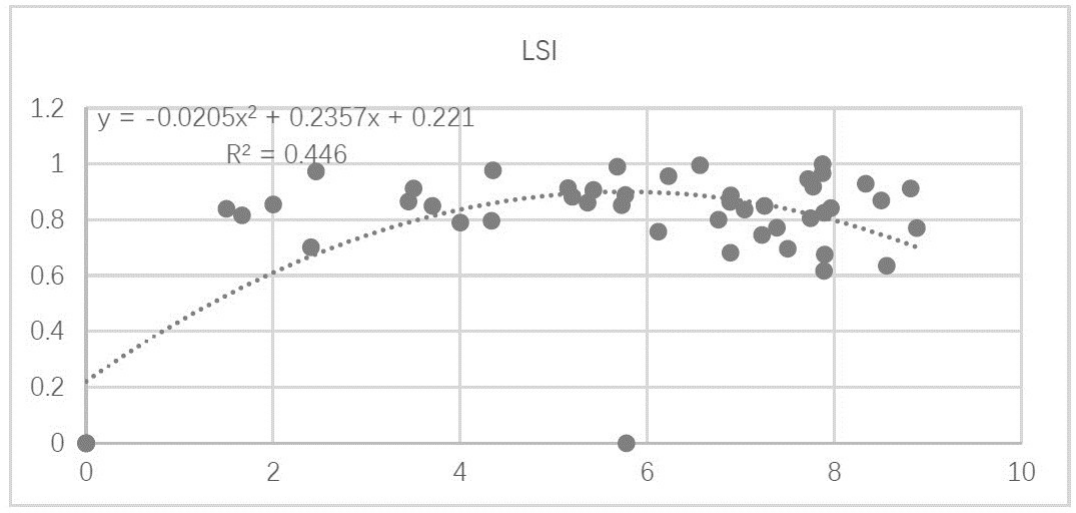
Relationship between landscape shape index (LSI) and forest ecosystem service function.

The relationship between LSI and forest ecosystem service function for the 53 study units is shown in Fig 9. With increasing LSI, forest ecosystem service function first increases exponentially, and reaches its highest value at LSI = 5, before decreasing gradually.

**Fig 10:**
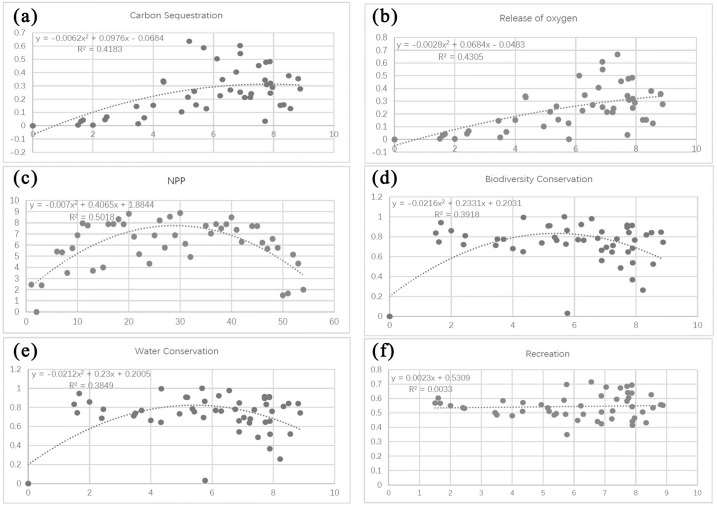
Relationships between the forest landscape shape index (LSI) and different forest ecosystem service functions. Plots show the relationships between LSI and (a) carbon sequestration capacity, (b) oxygen release capacity, (c) NPP capacity, (d) biodiversity conservation capacity, (e) water conservation capacity, and (f) recreation capacity.

Based on the fitted curve in the regression, the relationships between individual indicators of forest ecosystem service and the LSI index can be classified into three categories: (1) a significant exponential relationship, such as between LSI and carbon sequestration and oxygen release capacities (Fig 10A and 10B); (2) a second-order polynomial relationship, such as between LSI and the capacities for NPP, biodiversity conservation, and water conservation (Fig 10C–10E); and (3) no significant association, such as between LSI and recreational capacity (Fig 10F).

The first group of results demonstrate significant increases in carbon sequestration and oxygen release capacity with increasing forest shape complexity, which follow a clear exponential pattern. In particular, there is a rapid increase in capacities for both services when the LSI value approximates 5 (Fig 10A and 10B).

The second group of results show that as forest shape changes, the capacities for NPP, biodiversity conservation, and water conservation increase in a significant second-order polynomial relationship. The capacities for these three services increase significantly when LSI values range between 0 and 4, reach a maximum at LSI = 5, and gradually decrease over higher LSI values (Fig 10C–10E).

The third group of results have poor fit (i.e., R^2^ approximates zero). They indicate that recreational capacity varies irregularly with LSI, making it difficult to predict its changing behavior using a simple or segmented function (Fig 10F).

In sum, there is a clear quadratic polynomial relationship between the landscape shape index (LSI) and forest ecosystem service function. This may occur because as the survive vegetation in the forest increases in complexity and stability, it changes in shape, and has more exposure for gaseous exchange, thus enhancing forest ecosystem service capacity. When LSI reaches the critical value of 5, forest ecosystem service function reaches its highest value. However, when LSI exceeds 5, forest ecosystem service function declines; it may be that the forest topographic plate shape at these LSI levels is too complex, making it difficult to establish connections between vegetation communities and external gases, and leading to a decrease in biodiversity.

#### 4.3.3 Relationship between forest sprawl index (CONTAG) and forest ecosystem service function

**Fig 11:**
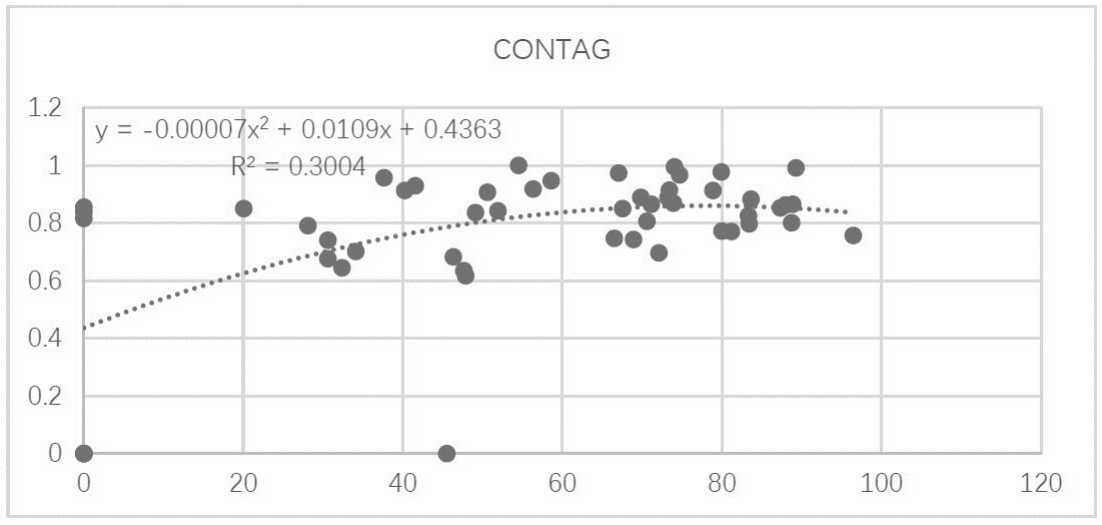
Relationship between the landscape sprawl index (CONTAG) and forest ecosystem service function.

The relationship between CONTAG and forest ecosystem service function for the 53 study units is shown in Fig 11. As values of CONTAG increase, forest ecosystem service function first increases exponentially, before peaking at CONTAG = 70, then decreasing gradually after.

**Fig 12:**
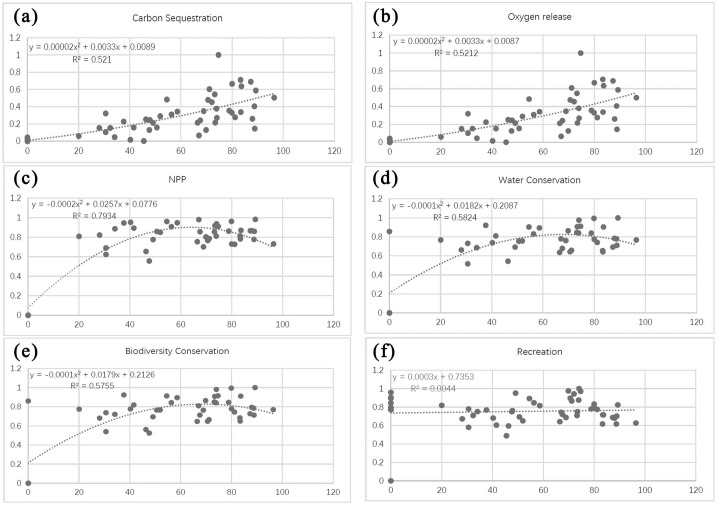
Relationships between the forest sprawl index (CONTAG) and different ecosystem service functions. Plots show the relationships between CONTAG and (a) carbon sequestration capacity, (b) oxygen release capacity, (c) NPP capacity, (d) water conservation capacity, (e) biodiversity conservation capacity, and (f) recreational capacity.

Based on the fitted curve in the regression, the relationships between individual indicators of forest ecosystem services and the CONTAG index can be classified under three categories: (1) a significant exponential relationship, such as between CONTAG and the capacities for carbon sequestration as well as oxygen release (Fig 12A and 12B); (2) a second-order polynomial relationship, such as between CONTAG and the capacities for NPP, water conservation, and biodiversity conservation (Fig 12C–12E); and (3) no significant association, such as between CONTAG and recreational capacity (Fig 12F).

The first group of results show a significant increase in capacities for carbon sequestration and oxygen release with increasing forest sprawl, which follow a clear exponential relationship. In particular, rapid increases in capacities for these two services were observed at CONTAG values approximating 70 (Fig 12A and 12B).

The second group of results show that as the forest sprawl changes, the capacities for NPP, water conservation, and biodiversity conservation also increase, and follow a significant second-order polynomial relationship. The capacities for these three services increase significantly at CONTAG values between 0 and 70, peaking at CONTAG = 70, before decreasing gradually at higher CONTAG values (Fig 12C–12E).

The third group of results have poor fit (i.e., R^2^ approximates zero). They indicate that recreational capacity varies irregularly with CONTAG, making it difficult to predict its changing behavior using a simple or segmented function (Fig 12F).

In sum, the results show that the sprawl index (CONTAG) has an obvious second-order polynomial relationship with forest ecosystem service function. Specifically, when CONTAG values increase—reflecting an increase in the degree of urban patchy agglomeration, a change in forest connectivity, and a gradual increase in urban forest sprawl—ecosystem service capacity gradually increases. When CONTAG reaches the critical value of 70, ecosystem service function achieves a degree of maximum stability, and gradually decreases at higher CONTAG values.

## 5 Discussion

### 5.1 Mechanisms linking landscape patterns and forest ecosystem service functions

A quantitative evaluation of forest ecosystem service functions is an important prerequisite for enhancing the stability and the utility of the ecological environment of forests. An understanding of the relationships between landscape patterns and forest ecosystem service functions allows for optimizing the construction and management of urban forests. It can also provide a basis for better urban planning, design, and resource allocation [80]. This study uses principles of landscape ecology and quantitative evaluations of forest ecosystem service functions in Renqiu, a typical pilot city of "National Forest City", to explore the relationships between landscape patterns and forest ecosystem service functions, and to identify solutions for urban green space allocation and the optimization of spatial structure. Combining qualitative and quantitative, so as to identify the problems of urban forest and improve urban green space construction, promote the logical transformation of urban green space construction, provide scientific theoretical support for the construction of national forest cities and ultimately provide theoretical basis and optimization strategies for the planning, construction, management and sustainable use of similar urban forests, so as to promote the sustainable and healthy development of urban forest ecology.

Previously, Ji et al. [81] investigated the value of forest ecosystem service functions in 101 sites in China, with a focus on forest area and forest stock. Wang et al. [28] analyzed forest landscape patterns of Ma’anshan City, using a combination of landscape indicators and gradient analysis to quantify urban spatial patterns. Wang et al. [82] investigated how the specific layout of landscape systems affected the stability of ecosystems in the Caijiachuan forest in the Huanglong Mountain forest area. Previous studies only used quantitative research and lacked the combination of quantitative and qualitative research.

The present study investigates the spatial and morphological impacts of forests on the values of forest ecosystem services values in addition to their stocks. It therefore expands on the abovementioned studies that used forest stocks as the only indicator of ecosystem services, and addresses forest structure and morphological indices. The results illustrate the importance of forest landscape patterns in terms of area, morphology, and structure for forest ecosystems. The correlation between the PLAND, LSI, and CONTAG indices and the overall as well as sub-services of forest ecosystems are further clarified. The results, which indicate that forests are the main providers of ecosystem services, are consistent with the Specification for the Assessment of Forest Ecosystem Services (GB/T 38582–2020) currently used in urban planning throughout China. moreover, the accuracy of the relationship is further enhanced by the use of accurate and up-to-date data from the study area of Renqiu City.

Overall, it appears that urban forest landscape patterns and their associated ecosystem services have a close causal feedback relationship. We find that not only is an increase in the percentage of forested area (PLAND) is positively correlated with the increase in ecosystem service capacity, but that the increase in ecosystem service capacity also reflects an increase in the formation and supply of major ecosystem services (e.g., carbon sequestration and oxygen release, NPP, water conservation, and biodiversity conservation). At the same time, changes in the landscape shape index (LSI) affect the flow and transmission of materials and energy in the forest, which not only affect the capacity for ecosystem-service formation and production, but which also make the distribution of ecosystem services in the landscape more spatially heterogeneous. Finally, an increase in the forest sprawl index (CONTAG) indicates better connectivity between forest patches; this begins to decline after a stable value is reached. This may occur because the increasing impacts of human activities on forests such as the control of the forest landscape start to decline, until a stable rate of development of in forests is reached. From the above indices, we contend that the landscape patterns of urban forests are intricately linked to the sustainability of ecosystem services, and therefore exert an important influence over the formation and supply of ecosystem services.

### 5.2 Strategies for the spatial optimization of forest in Renqiu City

From the quantitative evaluation of forest ecosystem service function in Renqiu City, as well as the observed correlation between forest landscape patterns and ecosystem service function, we identify several obstacles to the optimization of forest spaces for ecosystem services in Renqiu City. First, the overall fragmentation and heterogeneity of forest patches in the landscape is increasing. The landscape matrix of the study area comprises forest, which is the dominant land cover type in the study area. However, the forest landscape patches show a dispersed, fragmented and isolated distribution. Connectivity between the different forest patches is poor, and moreover, the patches are becoming increasingly complex in shape. Thus, as there is less clustering of forests, urban ecosystem service functions are likely to be impacted.

**Fig 13:**
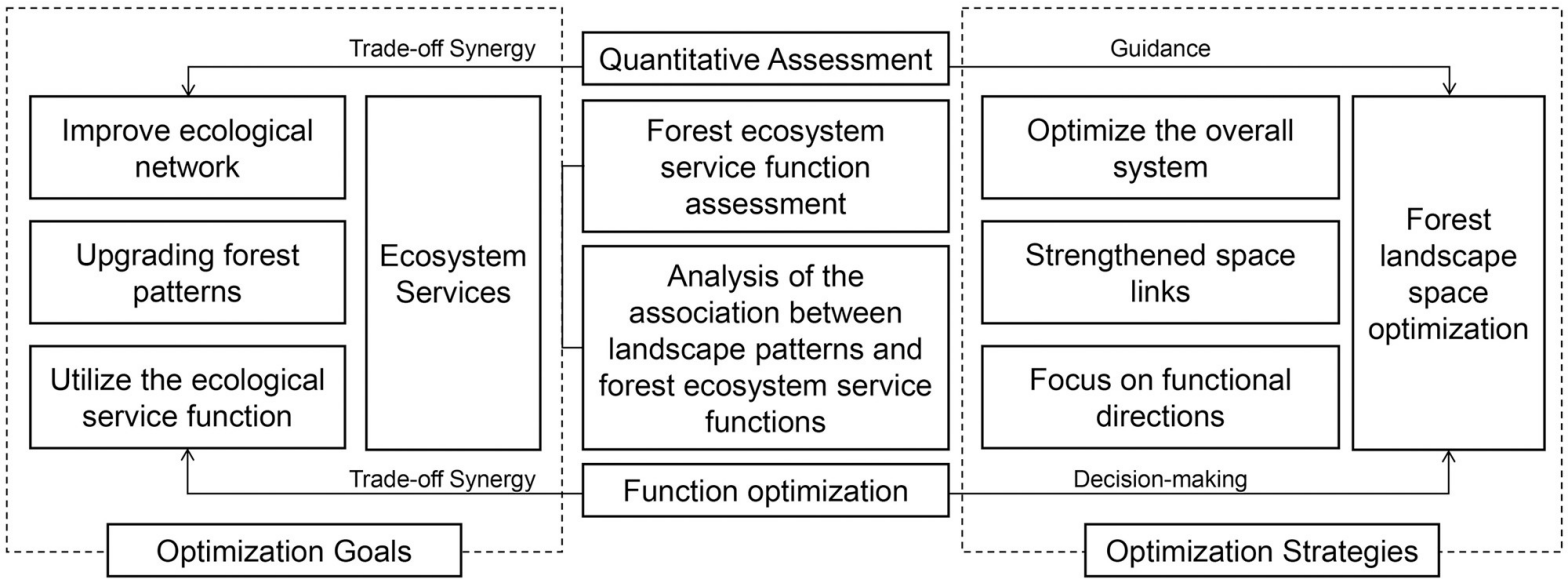
Relationships between forest ecosystem services and the spatial optimization of forest landscapes.

**Fig 14:**
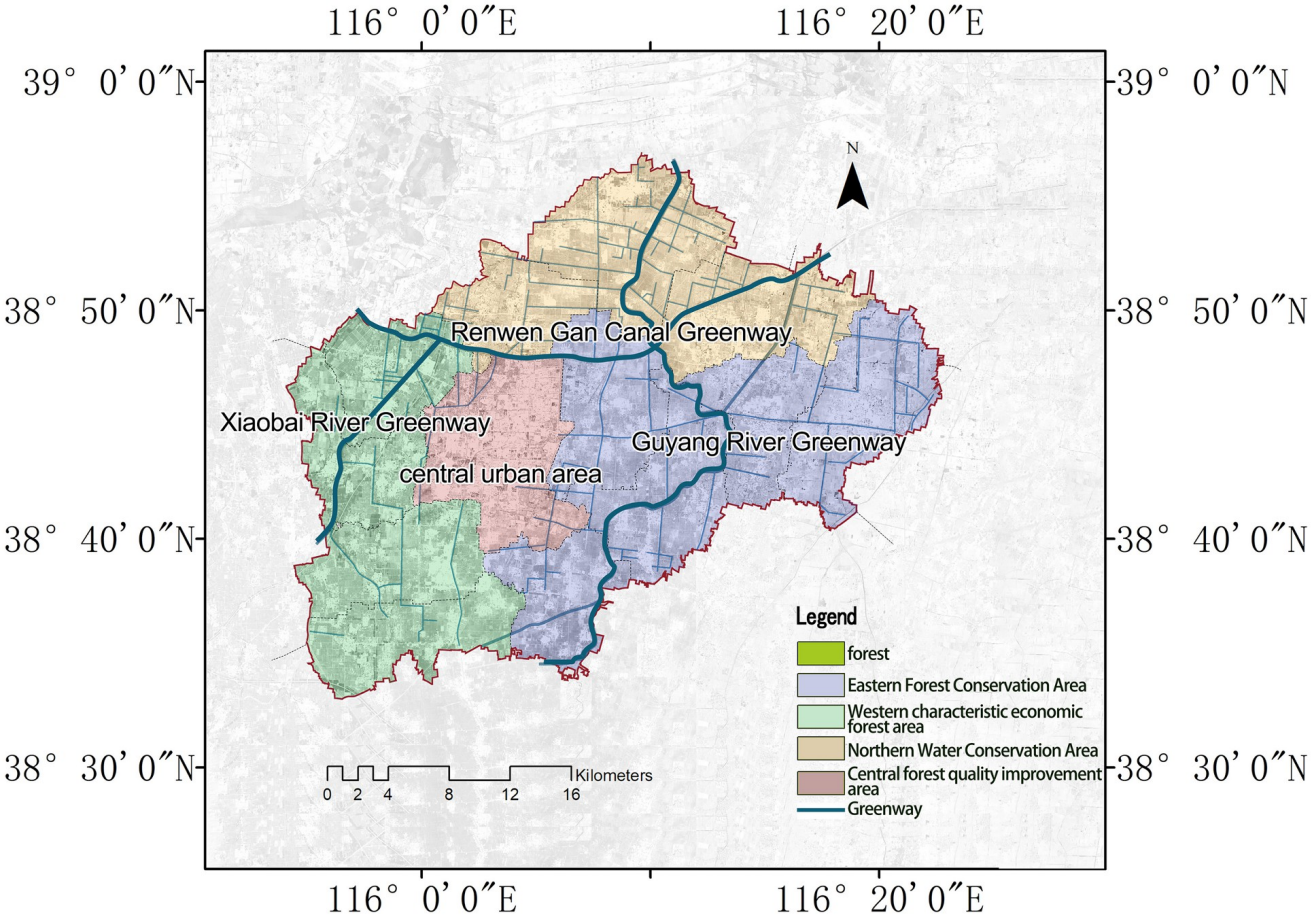
Diagram of spatial optimization strategy for forested landscape in Renqiu City.

To address these problems, we investigated ways to spatially optimize the forest landscape to optimize ecosystem services in Renqiu City. Specifically, we aimed to find ways to strengthen the connectivity between forest patches in space, upgrading forest patterns, and to propose strategies for spatial optimizing the forest landscape in Renqiu City from the perspective of creating a national forest city (Figs 13 and 14).

1. Optimize the overall forest ecosystem and improve ecological connectivity
  First, the restoration or construction of forested spaces should not only consider individual sites. Instead, such actions should be based on a broader perspective, as well as systematic and holistic planning, so as to improve the overall ecological connectivity among multiple forest patches as well as to ensure a reasonable and orderly development of urban forest landscape structure. Areas with sufficient green spaces—such as the northern areas as well as the southern parts of the central city—should adopt comprehensive strategies for improving the forest internal quality, such as by building self-sustaining near-natural communities. The forested areas of Renwen Gan Canal, Xiaobai River and Guyang River Basin are designated as the ecological red lines (The “ecological red line” is an aggregate minimum area where development is strictly controlled, ensuring the sustainable provision of ecosystem functions, environmental quality and resource usage). Adjacent to these, buffer zones should be put in place to control urban expansion, thus helping to mitigate fragmentation of the landscape and irrationally spaced landscape patches, while also protecting the forest matrix.
2. Strengthen spatial connectivity and enhance forest patterns
  Second, the dominant position of forest landscapes in Renqiu City should be protected. To enhance forest landscapes, managers can repair areas of degraded vegetation and of damaged abandoned land; this will help to improve and restore the ecological aspects of the forests. They can also work to increase forest coverage, help to maintain biodiversity by artificial afforestation, close hillsides to facilitate afforestation and improving forest quality; these actions will help to achieve the ecological advantages of forest resources. At the same time, different ecological patches such as forests and water bodies should be reasonably organized such that their specific functions can be optimized. The spatial links and connectivity between these patches should also be strengthened so as to promote the exchange of species, materials, energy and information within and between patches. This can be achieved by building municipal ecological corridors where forests and water networks are closely linked together. Examples of these include the “greenway” that exists on both sides of the Guyang River and its tributaries, as well as the Renwen trunk canal greenway and the Xiaobai River greenway. These greenways can be gradually promoted to become landscape types with simpler shapes and more complete patch structures, so as to enhance the spatial patterns of forests and to promote the sustainable development of urban forest ecosystems.
3. Focus on functional guidance and optimize ecosystem services
  Finally, the strategy of “functional guidance” from the perspective of ecosystem services is proposed for strengthening the dominant advantages, resolving the contradictions and enhancing the benefits of ecosystem stability. For areas where cultural services represent the dominant ecosystem service, such as in the Qingta Township and the Beixinzhuang Township adjacent to the Baiyangdian area, spatial connection can be further strengthened through appropriate forest management and the shaping of green space nodes, enhancing street greening, building small patches of forest near residential areas, connecting various forest patches in the city through urban greenways, increasing green space according to local conditions, strengthening social and cultural services within the area, and highlighting the dominant ecosystem service type. In the areas where regulation and support services are mainly guaranteed and enhanced, such as in the northern part of Renqiu City and the southeastern part of the central city, the regional focus should mainly emphasize improvements in the efficiency of regulation and support services. This can be achieved by focusing on preserving the quantity and improving the quality of forests, optimizing their configuration and layout, increasing the proportion of shrub forests, and building a green space system with a complex configuration of trees, shrubs and grasses.

## 6 Conclusion

Based on the analysis of multi-source data focusing on Renqiu City, Hebei Province, China, this study identifies the relationships between forest ecosystem service intensity and landscape pattern status, and proposes specific strategies for optimizing the urban forest ecosystems accordingly. The following conclusions were drawn:

1. The spatial distribution of forest ecosystem services in Renqiu City generally mirrors the spatial distribution of forests, and the intensity of ecosystem service functions increase with forest area. Based on the intensity of ecosystem functions, we can divide Renqiu into the northern Renwen Dry Drainage Basin, the southeastern Guyang River Basin, the western Xiaobai River Basin and the central urban area. Of these, the northern Renwen Dry Drainage Basin has the best ecosystemservice capacity, followed by the southeastern Guyang River Basin, the western Xiaobai River Basin and the central urban area, which has the weakest service capacity. In terms of forest ecosystem service capacity per unit area, the capacity of Z17, Z18, Z38, Z44, Z45, Z46, Z47 and Z52 are considerably higher than that of Z26, which is located in the center of Renqiu city.
2. From analyses of the mechanisms linking landscape pattern to forest ecosystem service function, the PLAND index shows a second-order polynomial relationship with forest ecosystem service function. Specifically, forest ecosystem service function increases with PLAND and has a critical PLAND value of 80. Forest ecosystem service function shows an increasing and then decreasing trend with increasing LSI; this is because increasing shape diversity affects the material exchange of the forest when forest shape is too complex. The CONTAG index has a second-order polynomial relationship with forest ecosystem service function and a critical value of 70; at this point, forest ecosystem service capacity stabilizes.
3. Assuming that optimizing landscape patterns help to optimize their associated ecosystem service functions, managers should consider the integrity of forest ecosystems, optimize their ability to self-succession, repair service functions of key nodes within forests, enhance forests’ structural stability, optimize forest quality and community structure, and strengthen the efficiency of functional transformation per unit area. As forest quality and community structure are optimized, and the efficiency of functional transformation per unit area should be enhanced, such that forest ecosystem service function can be in turn be maximized.

This study did not address the changes in forest ecosystem service functions over time due to data limitations; thus, further research in these areas is needed. As changes in forest ecosystem service functions are affected by a variety of factors, it is difficult to fully understand the relationships between forest ecosystem service functions and landscape patterns simply by relying on data and regression models. In practice, a variety of other factors such as stakeholders and human well-being must be considered; these aspects of forest ecosystem service functions shall be the focus of subsequent work.
